# Protein Arginine Methyltransferase 6 Involved in Germ Cell Viability during Spermatogenesis and Down-Regulated by the Androgen Receptor

**DOI:** 10.3390/ijms161226186

**Published:** 2015-12-10

**Authors:** Manling Luo, Yuchi Li, Huan Guo, Shouren Lin, Jianbo Chen, Qian Ma, Yanli Gu, Zhimao Jiang, Yaoting Gui

**Affiliations:** 1 Department of Physiology, Shantou University Medical College, Shantou 515041, China; lmnhunan@126.com (M.L.); 15096032050@163.com (Y.L.); 2 Guangdong and Shenzhen Key Laboratory of Male Reproductive Medicine and Genetics, Institute of Urology, Peking University Shenzhen Hospital, Shenzhen PKU-HKUST Medical Center, Shenzhen 518036, China; guohuan201405@163.com (H.G.); linshouren198710@126.com (S.L.); chenjianbo8090@163.com (J.C.); mq@whu.edu.cn (Q.M.); guyl2418@Chinaren.com (Y.G.); 13510857100@126.com (Z.J.); 3 Department of Surgery, Guangzhou Medical University, Guangzhou 510182, China; 4 Department of Surgery, Anhui Medical University, Hefei 230032, China

**Keywords:** PRMT6, androgen receptor, testes, migration, apoptosis

## Abstract

Androgens and the androgen receptor (AR) are of great importance to spermatogenesis and male fertility. AR knockout (ARKO) mice display a complete insensitivity to androgens and male infertility; however, the exact molecular mechanism for this effect remains unclear. In this study, we found that the expression levels of *Prmt6* mRNA and protein were significantly up-regulated in the testes of ARKO mice compared to wild type (WT) mice. PRMT6 was principally localized to the nucleus of spermatogonia and spermatocytes by immunofluorescence staining. Furthermore, luciferase assay data showed that AR together with testosterone treatment suppressed *Prmt6* transcription via binding to the androgen-responsive element (ARE) of the *Prmt6* promoter. Moreover, knockdown of *Prmt6* suppressed germ cells migration and promoted apoptosis. In addition, both of these cellular activities could not be enhanced by testosterone treatment. Taken together, these data indicate that PRMT6, which was down-regulated by AR and influenced cell migration and apoptosis of germ cells, could play a potentially important role in spermatogenesis.

## 1. Introduction

Androgens are critical steroid hormones that are responsible for the expression of the male phenotype, the accomplishment of sexual maturation, the maintenance of spermatogenesis as well as male reproductive function and behavior [1]. Androgens bind to the androgen receptor (AR) in cytoplasm, leading to AR transactivation and are translocated to the nucleus, then AR binds to the AREs (androgen response elements) on target genes, which leads to the regulation of AR downstream gene expression [2]. AR belongs to the nuclear receptor super family, mediates the biological action of androgens and regulates the expression of a number of androgen-responsive genes [1,3,4]. Therefore, in humans, aberrant androgens or AR actions are associated with multiple pathologies, such as androgen insensitivity syndromes, prostate cancer, testicular feminization, and male infertility [1,5]. However, the detailed molecular and cellular mechanisms regarding the mediation of these pathologies, specifically relating to spermatogenic cell development, by androgens and AR are not yet fully understood.

To search for the AR-regulated genes that are involved in spermatogenesis, transcriptional profiling studies of an AR knockout (ARKO) mouse model have been used. Using this mouse model, researchers have identified many candidate target genes of AR, such as Rhox5 [6], Tubb3 [7], and c-myc [8], but only Rhox5 has been characterized as a target of AR. Additional genes require further study to determine whether they are regulated by AR and are physiologically relevant to spermatogenesis.

The digital gene expression analysis data from our previous study [9] showed that protein arginine methyltransferase 6 (PRMT6) was one of 2865 genes expressed at a higher level in ARKO mice than wild-type (WT) mice, which prompted us to ask whether androgens and their receptor could regulate *Prmt6* expression. It was reported that PRMT2, one of the protein arginine methyltransferase family members, was recruited by and acted as a coactivator of AR in the presence of androgens [10]. Protein arginine methyltransferase 10 was also down regulated by AR [11]. Research also showed that PRMT6 could methylate and interact with AR, and that the interaction between them was obviously enhanced when AR was mutant [12]. Previous studies have demonstrated that PRMT6 interacts with AR [13] and have suggested that *Prmt6* is a non-obstructive azoospermia-susceptible locus [14]. Therefore, we hypothesized that *Prmt6* could be regulated by AR and play an important role in male reproduction.

PRMT6 is a type I arginine methyltransferase that predominantly resides in the nucleus and is highly expressed in human testes [15]. Previous studies have determined that PRMT6 methylates histone H3 at R2 in addition to H4R3 and H2AR3 [16,17]. PRMT6 was also observed to co-activate the transcription of estrogen, progesterone, and glucocorticoid receptor coupling with alternative splicing [18]. Microarray analysis on U2OS cells (a human osteosarcoma cells) revealed that cell migration and invasion were reduced in the absence of PRMT6 due to the activation of thrombospondin-1 [19]. It has also been determined that PRMT6 acts as an oncoprotein by directly binding to and repressing the p21 promoter, promoting the growth and prevents the senescence of breast cancer cells [20]. In addition, a study has shown that PRMT6 plays an important role as a regulator of DNA base excision repair by forming a complex with and methylating DNA polymerase β, which strongly stimulates DNA polymerase activity [21]. DNA base excision repair is a process that is highly efficient in human male germ cells, such as primary spermatocytes and round spermatids [22]. DNA polymerase β has also been found to participate in meiotic events during synapsis and recombination [23]. Accordingly, Hu *et al.* [24] suggested that PRMT6 could have function in synapsis and recombination through meiosis by regulating DNA polymerase β.

To date, however, little has been reported about PRMT6 relative to male reproduction. The aim of this study was to explore the expression pattern and function of PRMT6 during mouse spermatogenesis. We found that *Prmt6* mRNA and protein expression were significantly up-regulated in the testes of ARKO mice. Conversely, *Prmt6* transcription was suppressed by AR along with testosterone treatment. Furthermore, knockdown of PRMT6 functioned to repress cell migration and to promote cellular apoptosis; however, both these cellular activities could not be enhanced by testosterone treatment though AR could down-regulate *Prmt6* expression. Taken together, our data suggested that PRMT6 was down-regulated by AR and could be involved in spermatogenesis.

## 2. Results

### 2.1. Prmt6 (Protein Arginine Methyltransferase 6) mRNA and PRMT6 Protein Expression Were Increased in the Testes of ARKO (Androgen Receptor Knockout) Mice

**Figure 1:**
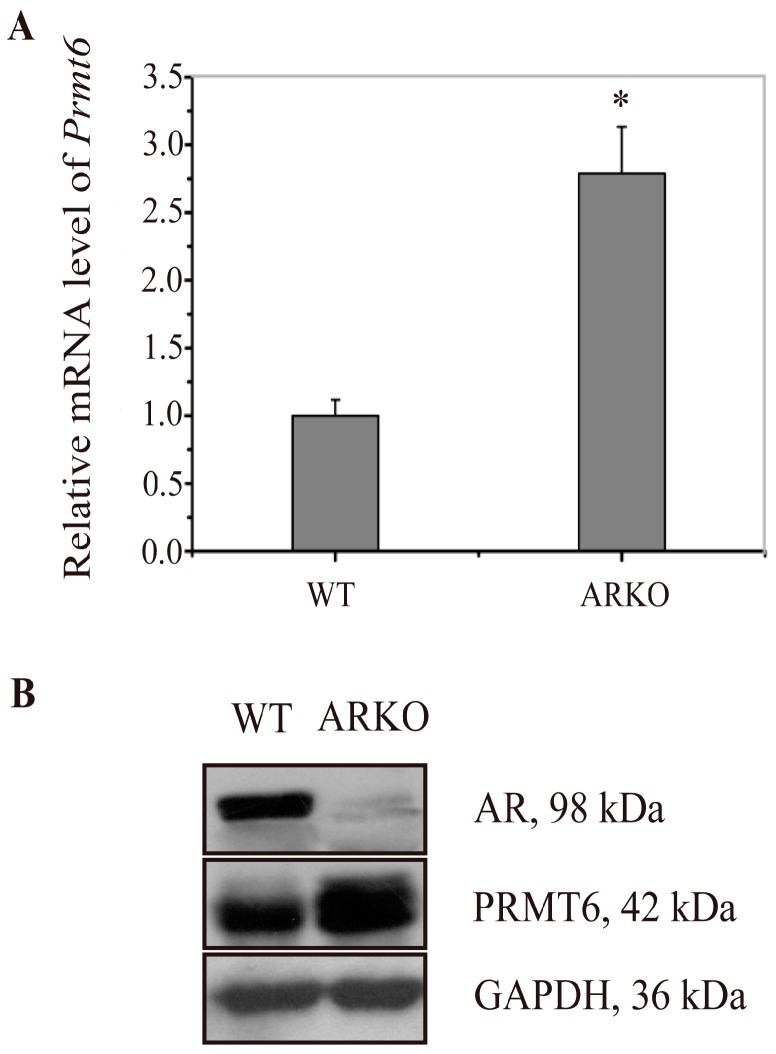
Increased expression of *Prmt6* (protein arginine methyltransferase 6) mRNA and PRMT6 protein in the testes of ARKO (androgen receptor knockout) mice. (**A**) The expression of *Prmt6* mRNA in the WT (wild type) and ARKO mouse testes were examined by RT-qPCR. The mean values of WT *Prmt6* mRNA was set as 1, and ARKO *Prmt6* mRNA was increased to 2.74 when compared with WT. Data were expressed as the mean ± SD (* *p* < 0.05, *n* = 5); (**B**) Representative graphs of the western blotting results for the expression of PRMT6 protein in the testes of WT and ARKO mice. GAPDH (glyceraldehyde-3-phosphate dehydrogenase) was used as an internal control. (*n* = 5).

To verify our previous digital gene expression analysis results, the expression of *Prmt6* mRNA and PRMT6 protein were examined by RT-qPCR (reverse transcription and quantitative real-time polymerase chain reaction) and western blotting in the testes of WT and ARKO mice. Compared with WT mice, *Prmt6* mRNA was increased to 2.74-fold in ARKO mouse testes (Figure 1A), and this finding was confirmed at the protein level by western blotting (Figure 1B). The results showed that the expression of *Prmt6* mRNA and PRMT6 protein were increased in ARKO mice, thus we speculate that PRMT6 was probably regulated by AR.

### 2.2. The Expression of Prmt6 mRNA and PRMT6 Protein Localization during Mouse Testes Development

**Figure 2:**
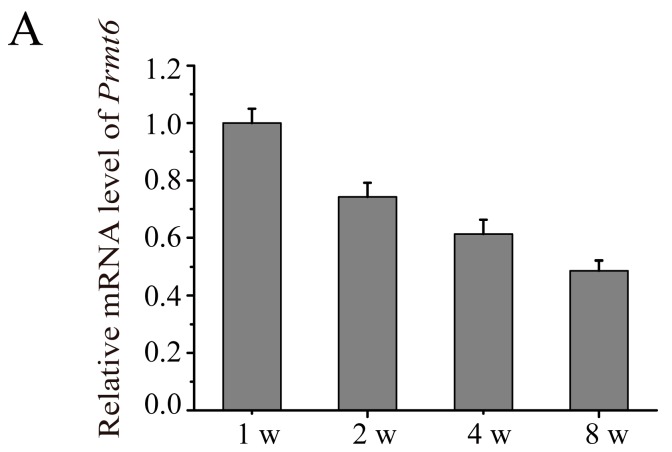

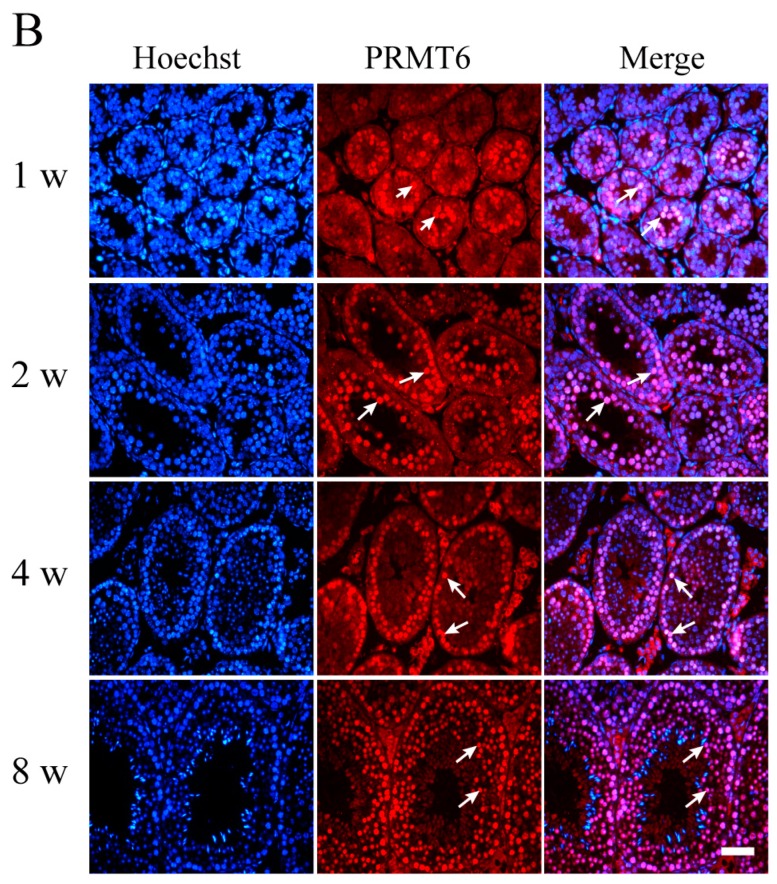
The expression of *Prmt6* mRNA and PRMT6 protein during mouse testes development. (**A**) RT-qPCR analysis was used to detect *Prmt6* mRNA in developing postnatal mouse testes. The relative expression of *Prmt6* at different stages of testes development was compared to one week (w). *Gapdh* was used as an internal control. Data are expressed as the mean ± SD (*n* = 5); (**B**) Immunofluorescence analysis was used to determine the localization of PRMT6 in mouse testes using a PRMT6 antibody (red). The nuclei of cells were labeled with Hoechst 33342 (blue). Purple represented the merging color of red and blue. Arrow: spermatogonia or spermatocytes. Bar = 50 μm.

RT-qPCR and immunofluorescence staining were used to examine the expression pattern of *Prmt6* during testes development. As shown in Figure 2A, *Prmt6* was expressed at all stages of testicular development and showed a decreasing trend from 1–8 weeks of postnatal development. Our immunofluorescence staining results revealed that PRMT6 was predominately located in the nucleus of spermatogonia and spermatocytes from 1–8 weeks of development, and little expression of PRMT6 was observed in Leydig and Sertoli cells (Figure 2B).

### 2.3. Sub-Cellular Localization of the EGFP (Enhanced Green Fluorescent Protein)-PRMT6 Fusion Protein

**Figure 3:**
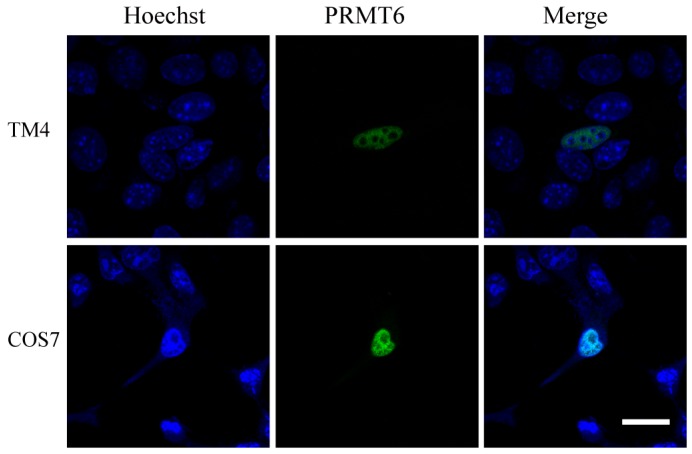
Sub-cellular localization of PRMT6 in TM4 and COS7 cells. The recombinant EGFP-PRMT6 plasmid was transiently transfected into TM4 and COS7 cells. The EGFP-PRMT6 protein (green) was expressed in TM4 and COS7 cells. The nuclei of the cells were stained with Hoechst 33342 (blue). Bar = 20 μm.

A pEGFP-C1-PRMT6 fusion plasmid was transiently transfected into TM4 and COS7 cells to detect the expression of the EGFP-PRMT6 fusion protein. As shown in Figure 3, the EGFP-PRMT6 protein was primarily detected in the nuclei of both TM4 and COS7 cells. This nuclear localization suggested that PRMT6 might be involved in the regulation of nuclear processes.

### 2.4. In Vitro Repression of Prmt6 Promoter Activity by Androgen Receptor (AR) and Testosterone

**Figure 4:**
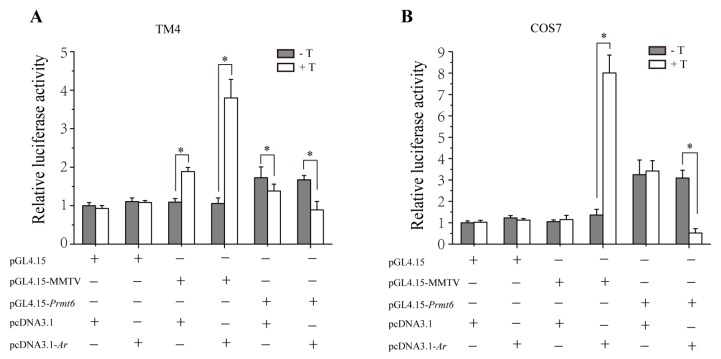
Inhibition of *Prmt6* promoter activity by AR along with testosterone treatment in TM4 and COS7 cells. (**A**,**B**) TM4 and COS7 cells were transiently cotransfected with 200 ng of pGL4.15 (which has a firefly luciferase reporter gene) or pGL4.15-MMTV (mouse mammary tumor virus long terminal repeat, which was a hormone responsive promoter and located in the upstream of the luciferase reporter gene) or pGL4.15-*Prmt6* (containing the promoter region of *Prmt6*), 200 ng of pcDNA3.1 (empty vector) or pcDNA3.1-*Ar* (over-expression of *Ar*, containing the coding sequence of *Ar*) and treated with or without testosterone (T, 10 nM). The relative luciferase activity was calculated from firefly luciferase data standardized to renilla luciferase data. All the experiments were performed in triplicate and repeated at least three times. Data are expressed as the mean ± SD. * *p* < 0.05.

To evaluate whether AR and testosterone (T) affects the transcription of *Prmt6*, we used luciferase assay to measure the *Prmt6* promoter-driven luciferase activity. As shown in Figure 4, both in TM4 and COS7 cells, when pGL4.15 was cotransfected with pcDNA3.1 or pcDNA3.1-*Ar* as negative controls, there was no difference of the luciferase activity between cells treated with and without T. As positive controls, the luciferase activity driven by pGL4.15-MMTV was significantly increased in the presence of pcDNA3.1-*Ar* and T. Meanwhile compared with pGL4.15 and pGL4.15-MMTV, the luciferase activity driven by *Prmt6* promoter was increased when cotransfected with pcDNA3.1 without T. However, the luciferase activity driven by pGL4.15-*Prmt6* was significantly reduced in the presence of AR and T. Because TM4 cells have endogenous AR expression, compared with that without T, the luciferase activity also had an increase when pGL4.15-MMTV was cotransfected with pcDNA3.1 in the presence of T; oppositely, a decrease was found in pGL4.15-*Prmt6* under the same condition. No difference was observed in the luciferase activity between COS7 cells treated with and without T when pcDNA3.1 was cotransfected either with pGL4.15-MMTV or pGL4.15-*Prmt6*. Taken together, the above data indicated that AR and testosterone probably repressed the promoter activity of *Prmt6*.

### 2.5. The Expression of Prmt6 mRNA and PRMT6 Protein Were Regulated by AR in Vitro

**Figure 5:**
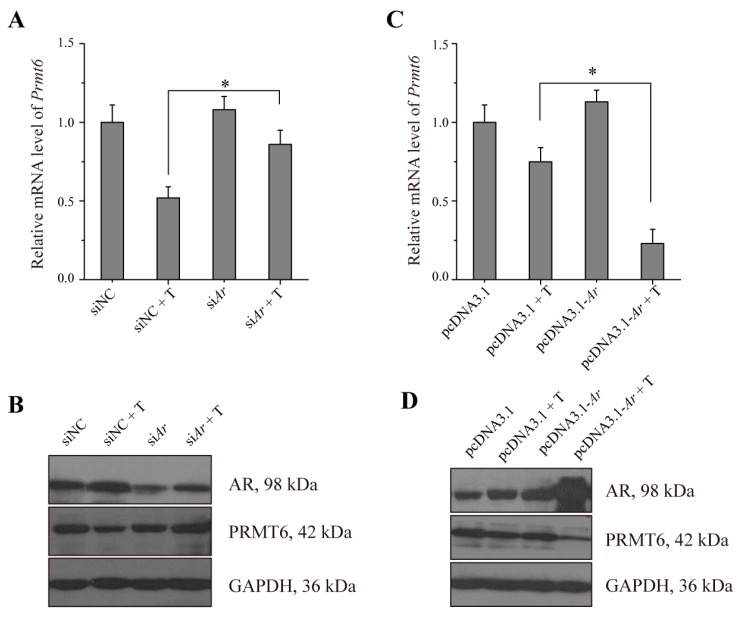
The expression of *Prmt6* mRNA and PRMT6 protein were down-regulated by AR in TM4 cells. (**A**,**B**) TM4 cells were transiently transfected with siNC or si*Ar*, and treated with or without 10 nM testosterone (T), then the expression of *Prmt6* mRNA and PRMT6 or AR protein were detected by RT-qPCR and western blotting, respectively. All of the experiments were repeated at least three times. Data are expressed as the mean ± SD. * *p* < 0.05; (**C**,**D**) TM4 cells were transiently transfected with pcDNA3.1 or pcDNA3.1-*Ar* plasmid and treated with or without 10 nM testosterone. RT-qPCR and western blotting were used to examine the expression of *Prmt6* mRNA and PRMT6 or AR protein, respectively. All of the experiments were repeated at least three times. Data are expressed as the mean ± SD. * *p* < 0.05.

To further confirm that AR regulates *Prmt6* mRNA and PRMT6 protein expression, TM4 cells were employed. Our results showed that in siNC (non-targeting siRNA, set as negative control) transfected cells the expression of *Prmt6* was decreased when treated with testosterone. Transfection with si*Ar* (siRNA of *Ar*) along with testosterone treatment resulted in an increased expression of *Prmt6* when compared with siNC and testosterone (Figure 5A). Meanwhile the expression of *Prmt6* was decreased in cells treated with pcDNA3.1-*Ar* together with testosterone treatment (Figure 5C). Western blotting analysis confirmed our RT-qPCR results (Figure 5B,D). These data supported the hypothesis that *Prmt6* mRNA and PRMT6 protein were probably regulated by AR.

### 2.6. The Knockdown of Prmt6 Represses Germ Cell Migration

**Figure 6:**
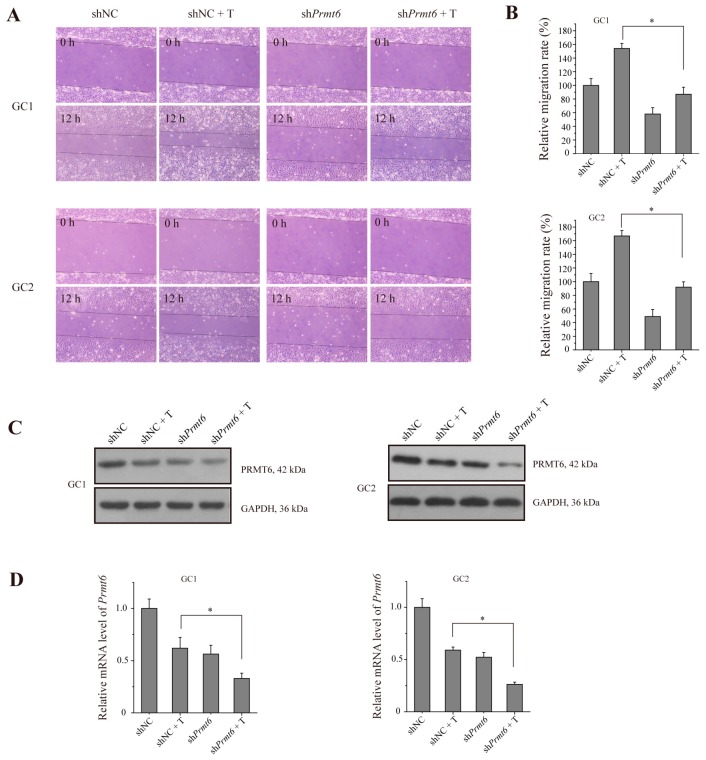
The migratory ability of germ cells is repressed by blocking the function of PRMT6 together with testosterone (T) treatment. Cells were transfected with shNC or sh*Prmt6* together with or without 10 nM testosterone treatment. (**A**) Representative photographs of migration assay in GC-1 and GC-2 cells on 0 h (represented the time when the scratch was made) and 12 h are shown; (**B**) Statistical analysis of the relative migratory ability of GC-1 and GC-2 cells; (**C**,**D**) The expression of PRMT6 protein and *Prmt6* mRNA in GC-1 and GC-2 cells were examined by western blotting and RT-qPCR, respectively. The expression of *Gapdh* mRNA and GAPDH protein were used as a loading control. All of the experiments were repeated at least three times. Data are expressed as the mean ± SD. * *p* < 0.05.

To determine whether PRMT6 expression was of functional significance during spermatogenesis, we performed a migration assay on germ cells. As shown in Figure 6A, both sh*Prmt6* (shRNA of *Prmt6*)-treated GC-1 and GC-2 cells showed a significantly shorter migration distance compared to shNC (non-targeting shRNA, set as negative control)-treated control cells. As shown in Figure 6B, the relative migration rate was significantly different between sh*Prmt6*- and shNC- along with testosterone treatment cells. These results also showed that the migration of sh*Prmt6*- together with testosterone treatment was not reduced when compared to sh*Prmt6*-treated group. PRMT6 protein and *Prmt6* mRNA expression were detected in both cell types by western blotting and RT-qPCR, respectively, and the results showed that both PRMT6 protein and *Prmt6* mRNA expression were decreased when treated with testosterone or sh*Prmt6*; testosterone together with sh*Prmt6*-treated cells showed a lower expression (Figure 6C,D). Thus, PRMT6 could play an important role in germ cell migration. However, testosterone treatment promotes cell migration. As a consequence, reduced PRMT6 expression by sh*Prmt6* together with testosterone treatment could not enhance the suppression of cell migration activity when compared with sh*Prmt6*-treated cells.

### 2.7. The Down-Regulation of Prmt6 Induces Germ Cell Apoptosis

**Figure 7:**
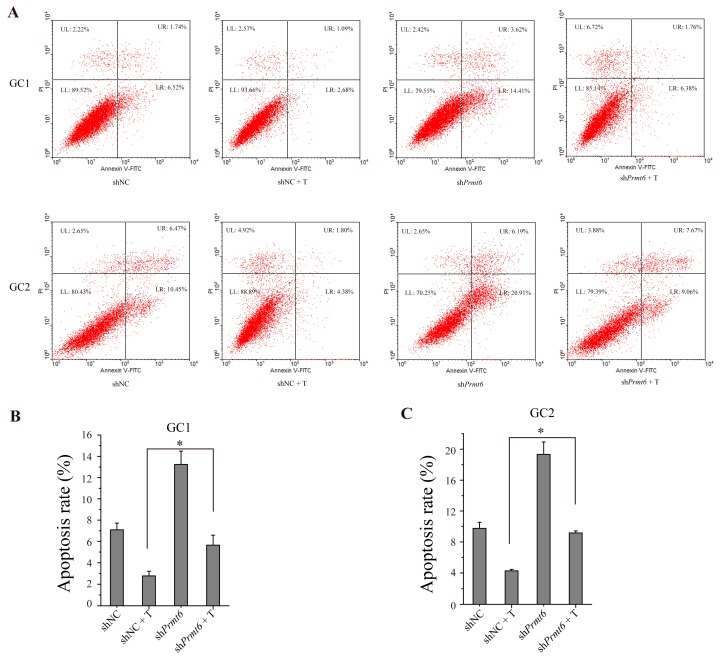
Germ cell apoptosis was promoted by transfecting GC-1 and GC-2 cells with sh*Prmt6* and treated with testosterone (T). (**A**) Representative graphs of GC-1 and GC-2 cell apoptosis as analyzed by flow cytometry. LR (lower right section of the graphs) which was Annexin V-FITC positive and PI (propidium iodide) negative represented the percentage of apoptosis cells; (**B**,**C**) Statistical analysis of the cell apoptosis rate of GC-1 and GC-2 cells. All of the experiments were repeated at least three times. Data are expressed as the mean ± SD. * *p* < 0.05.

We used flow cytometry to determine the effects of PRMT6 on germ cell apoptosis. As shown in Figure 7A, the rates of apoptosis were higher in GC1 and GC2 cells transfected with sh*Prmt6* compared to shNC-transfected controls. Statistical analysis (Figure 7B,C) showed that the apoptosis rate between sh*Prmt6* and shNC together with testosterone treatment was significantly different in these two cell lines. The results also showed that the apoptosis was not enhanced by sh*Prmt6* along with testosterone treatment when compared with sh*Prmt6*-treated cells. Thus, PRMT6 could play an important role in germ cell apoptosis, and AR together with testosterone treatment might have other crucial roles to affect germ cell apoptosis besides down-regulation *Prmt6* expression.

## 3. Discussion

Androgens/androgen receptor signaling is an important pathway for spermatogenesis and male fertility. Patients with AR mutations exhibit abnormal spermatogenesis and male sterility [25,26]. Male total ARKO mice show a typical female external appearance, and their testes are located abdominally with severely disrupted germ cell development. This phenotype is similar to human androgen insensitivity syndrome and testicular feminization [3]. Although there is extensive knowledge about the phenotypic consequences of *Ar* mutations, the exact mechanism has remained less clear. A previous study that transcriptionally profiled AR mutants revealed that more transcripts were up-regulated than down-regulated, which highlighted the role of AR as a transcriptional repressor in the testes [27]. In the present study, we supposed that AR down-regulated the expression of *Prmt6* mRNA and PRMT6 protein and found that the expression of *Prmt6* mRNA and PRMT6 protein was significantly up-regulated in the testes of ARKO mice.

When we verified the relationship between *Prmt6* and *Ar in vitro*, we found that the results were in agreement with previous *in vivo* results [9]. The expression of *Prmt6* mRNA and PRMT6 protein was increased when *Ar* was knocked down in TM4 cells, and the over-expression of AR led to a decrease in *Prmt6* mRNA and PRMT6 protein expression. Furthermore, we found that activated AR by testosterone-binding could in turn repress the activity of the *Prmt6* promoter. All of these findings support the hypothesis that AR regulates the function of PRMT6. In general, androgens and their receptor affect the development of spermatogenic cells [3,28]. Our experiments showed that the distribution of PRMT6 was almost exclusively localized to the nuclei of spermatogonia and spermatocytes. Thus, PRMT6 could be down-regulated by androgens and AR and consequently influence spermatogenesis.

We also found that *Prmt6* expression showed a trend towards decreased expression over time from 1–8 weeks in postnatal testes. These observations together with the distribution of PRMT6 supported a functional role of PRMT6 in the process of spermatogenesis, likely in the regulation of the process of meiosis. However, the mechanism by which PRMT6 influences spermatogenesis has not been reported. Some publications have helped to shed light on the major function of PRMT6. For instance, it has been shown that PRMT6 can be recruited to chromatin and function as a co-activator with nuclear factor-κB to facilitate transcription [29]. Other results have established that PRMT6 is more highly expressed in cancer cells than in non-neoplastic cells [30,31]. In addition, another study showed that knocking down PRMT6 resulted in cell proliferation defects [32]. Therefore, we hypothesized that PRMT6 could contribute to cell proliferation during spermatogenesis.

In our current study, we found that interfering with the function of PRMT6 using shRNA significantly inhibited the migration of germ cells, which suggested that PRMT6 could facilitate the proliferation of spermatogenic cells and serve a crucial function in the meiosis process. In addition to cellular proliferation, a previous study also showed that PRMT6 mediated cigarette smoke extract-induced apoptosis in human umbilical vein endothelial cells, in which the PRMT6 protein showed a decreased level [33]. PRMT4 (also known as CARM1), another protein arginine methyltransferase family member, also could inhibit cell proliferation and induce apoptosis when it was knocked down by siRNA [34]. In our present study, knocking down PRMT6 expression promoted the apoptosis of germ cells, suggesting that PRMT6 could also play a role in mediating apoptosis of spermatogenic cells. Taken together, these data suggest that knockdown of PRMT6 could block cell proliferation and promote apoptosis in spermatogenic cells. We also found that testosterone could facilitate cell migration and reduce cell apoptosis. The effects of testosterone, however, was so complex that lower expression level of PRMT6 by sh*Prmt6* together with testosterone treatment had a bigger cell migration effect than sh*Prmt6* treatment. Similarly to migration, the results of apoptosis were not enhanced by sh*Prmt6* together with testosterone treatment when compared with sh*Prmt6*-treated cells. In the testes of ARKO mice, spermatogenesis is severely arrested and apoptosis is increased [3,28]. However, these mice also show high levels of PRMT6, which could be the result of a compensatory mechanism aiming to reverse the low rate of proliferation and high level of cell apoptosis in the ARKO mouse testes. While the androgens/androgen receptor signaling was very complex, only the function of PRMT6 cannot efficiently inhibit the severe defects on ARKO mice.

## 4. Experimental Section

### 4.1. Animals and Samples

The ARKO mice used in this study were obtained from the Model Animal Research Center of Nanjing University (Nanjing, China). C57BL/6 mice were purchased from the Animal Center of Southern Medical University (Guangzhou, China). All animals were treated according to the Guide for the Care and Use of Laboratory Animals prepared by the Institute of Laboratory Animal Resources for the National Research Council. The study was endorsed by the ethics committee of Peking University Shenzhen Hospital (Permit Number: 2011-002, 11 May 2011). ARKO mouse testes were collected from adult ARKO mice and postnatal testes were individually collected from C57BL/6 mice aged 1–8 weeks. Other organs, such as the epididymis, bladder, kidney, liver, spleen, lung, heart and brain, were all collected from C57BL/6 adult mice.

### 4.2. Cell Culture

COS7 [7] (which has an easy cultured and transfected character, and expresses the large T-antigen), TM4 (which is derived from testes, and expresses AR) and germ cell lines (GC-1 and GC-2 cells, representing immortalized spermatogonia and spermatocytes, respectively; GC1 and GC2 cells with a high expression level of PRMT6) were obtained from ATCC (the American Type Culture Collection) and cultured in DMEM (Life Technologies, Rockville, MD, USA) supplemented with 10% FBS (fetal bovine serum; Gibco, Carlsbad, CA, USA) and penicillin-streptomycin (100 U/mL penicillin and 100 μg/mL streptomycin; Gibco, Carlsbad, CA, USA). All cells were maintained in a humidified atmosphere containing 95% air and 5% CO_2_ at 37 °C.

### 4.3. Total RNA Isolation and Quantitative Real-Time RT-PCR (RT-qPCR)

Total RNA isolation and RT-qPCR reactions were carried out as described previously [35]. The primers for mouse *Prmt6* were as follows: 5′-TGCCTACCTGTGCTTCCTTA-3′ (forward) and 5′-CTCCTGTCACTCTCAGAATTGC-3′ (reverse) (product size 183 bp). The primers for *Ar* were 5′-ACCTCTTCTTCCTGGCATACT-3′ (forward) and 5′-TCACTCTCCTGGCTTGTCA-3′ (reverse) (product size 155 bp). *Gapdh* was used as an internal control, and the primers for *Gapdh* were 5′-AGTGGCAAAGTGGAGATT-3′ (forward) and 5′-GTGGAGTCATACTGGAACA-3′ (reverse) (product size 83 bp). The relative quantification of target gene expression was estimated by the Applied Biosystems comparative Ct method (2^−ΔΔ*C*t^ Method).

### 4.4. Western Blotting Analysis

Proteins were extracted from ARKO mouse testes, different tissues of WT adult mice, TM4 and germ cells by using RIPA Lysis Buffer (Beyotime Biotechnology, Shanghai, China) supplemented with a protease inhibitor cocktail (Sigma, Spruce Street, St. Louis, MO, USA), and the protein content was quantified by using the Pierce™ BCA Protein Assay Kit (No. 23227; Thermo Scientific, Meridian Rd., Rockford, IL, USA). Then, the proteins were subjected to 10% sodium dodecyl sulfate-polyacrylamide gel electrophoresis (SDS-PAGE) and transferred onto polyvinylidene difluoride membranes (PVDF) (ImmobilonP, 0.45 μm-pore-size; Millipore, Bedford, MA, USA). Prior to being incubated with a mouse anti-PRMT6 antibody (1:1000; Cell Applications, San Diego, CA, USA), rabbit anti-AR antibody (ab74272, 1:1000; Abcam, Cambridge, MA, USA), or mouse anti-GAPDH antibody (1:1000; ZSGB-BIO, Beijing, China) overnight at 4 °C, the membrane was blocked in TBS-T (Tris-buffered saline plus 0.2% Tween 20) containing 5% nonfat milk for 1 h at room temperature. After being washed three times in TBS-T, the membrane was incubated in the appropriate secondary antibodies conjugated with horseradish peroxidase (HRP) (1:5000; Abgent, San Diego, CA, USA) for 1 h at room temperature. After an additional three washes in TBS-T to rinse the secondary antibodies, the proteins were detected by using a Chemiluminescence Phototube-HRP kit (WBKLS0500; Millipore, Bedford, MA, USA).

### 4.5. Immunofluorescent Localization of PRMT6

Mouse testes were fixed in 4% paraformaldehyde, embedded in paraffin, and cut in 3-μm sections onto poly-l-lysine-coated slides. The paraffin embedded testicular tissue was de-waxed and rehydrated before being subjected to antigen retrieval via immersion in 10 mM sodium citrate (pH 6.0) and microwaving for 30 min at 1000 W. After being cooled to room temperature, the sections were blocked in 10% BSA (bovine serum albumin) for 30 min at 37 °C. Then, the sections were incubated with a mouse anti-PRMT6 antibody (diluted 1:100 with PBS; Cell Applications, San Diego, CA, USA) overnight at 4 °C. The sections were washed in PBS three times followed by incubation with an anti-mouse-Alexa Fluor 594 antibody (diluted 1:500; Invitrogen, Carlsbad, CA, USA) for 1 h at room temperature. The slides were washed with PBS and stained with Hoechst 33342 (diluted 1:2000; Invitrogen) for 5 min at room temperature. Following two times of additional washes, the sections were mounted with SlowFade (Invitrogen), and the sections were viewed under a fluorescent microscope LEICADM4000B (Zeiss, Thuringia, Germany).

### 4.6. Construction and Sub-Cellular Localization of EGFP-PRMT6 Fusion Vector

To detect the sub-cellular localization of PRMT6 protein, we generated a fusion protein of PRMT6 with enhanced green fluorescent protein. The coding sequence of mouse *Prmt6* was amplified by PCR using the following primers: 5′-CCGGAATTCTATGTCGCTGAGCAAGAAAAGAAAGC-3′ (introducing a EcoRI site in 5′), 5′-CGCGGATCCTCAGTCCTCCATGGCAAAGTCT-3′ (introducing a BamHI site in 5′), and the PCR products were double-digested by restriction endonucleases of EcoRI and BamHI (Takara Biotechnology Co., Ltd., Otsu, Japan). Subsequently, the digested products were sub-cloned into the EcoRI and BamHI sites of the pEGFP-C1 vector to produce a pEGFP-C1-PRMT6 fusion protein expression vector, and the coding sequence of EGFP-PRMT6 was confirmed by DNA sequencing. The recombinant plasmid was transfected into COS7 cells and TM4 cells using Lipofectamine 2000 (Invitrogen) according to the manufacturer’s instructions. After a 48-h transfection, COS7 cells and TM4 cells were stained with Hoechst 33342 (Invitrogen), and the sub-cellular localization of GFP-PRMT6 in the treated cells was observed by fluorescent microscopy (LEICA DM4000B, Zeiss).

### 4.7. Plasmid Constructs and Luciferase Assay

The *Prmt6* promoter was amplified with the primers 5′-CGGGGTACCGAGATTACAAGTACA-GCTGAGGATT-3′ (forward) and 5′-CTAGCTAGCTCGTTGCGCGGTGC-3′ (reverse) from the mouse genome. The PCR product was sub-cloned into pGL4.15 at KpnI/NheI sites, and the sequence of the clone was verified by DNA sequencing. TM4 and COS7 cells (1.5 × 10^4^/well) were seeded in 24-well plates for approximately 24 h and then cotransfected with 200 ng of pGL4.15 (which has a firefly luciferase reporter gene) or pGL4.15-MMTV (mouse mammary tumor virus long terminal repeat, which was a hormone responsive promoter and located in the upstream of the luciferase reporter gene) or pGL4.15-*Prmt6* firefly luciferase reporter plasmid, 20 ng of pRL-TK renilla control luciferase reporter plasmid and 200 ng of pcDNA3.1 or pcDNA3.1-*Ar* (over-expressing of *Ar*). Six hours after transfection, the medium was replaced, and the cells were treated with or without 10 nM testosterone (T) before examining by luciferase assay. The Dual-Luciferase Reporter assay system (Promega, Madison, WI, USA) was used to measure the firefly and renilla luciferase activities. The relative luciferase activity was calculated from firefly luciferase data standardized to the renilla luciferase data. All of the experiments were performed in triplicate and repeated at least three times.

### 4.8. RNA Interference

To investigate whether AR regulates *Prmt6* mRNA and PRMT6 protein expression, siRNA against *Ar* (si*Ar*): 5′-GGGCAAUUCAACCAUAUCUTT-3′ [36] was used and transfected into TM4 cells. The sequence of siNC, 5′-UUCUCCGAACGUGUCACGUTT-3′, was used as a negative control. According to the manufacturer’s protocols, 3–5 × 10^5^ TM4 cells were seeded in a 6-well plate for approximately 24 h and then transfected with 30 pmol si*Ar* or siNC together with 9 μL Lipofectamine^®^ RNAiMAX Reagent (Invitrogen) per well. Six hours after transfection, the cells were treated with or without 10nM testosterone. After 48 h, RNA and protein were extracted from these cells, respectively, and used for next experiments.

### 4.9. Short Hairpin RNA-Mediated Knockdown

To detect the functions of PRMT6 *in vitro*, we used short hairpin RNA to interfere with the expression of PRMT6. The siRNA sequences against mouse *Prmt6* (sense: 5′-CACCGTGGAAAGCATGTAGTATAATTCAAGAGATTATACTACATGCTTTCCATTTTTTG-3′ and antisense: 5′-GATCCAAAAAATGGAAAGCATGTAGTATAATCTCTTGAATTATACTACA-TGCTTTCCAC-3′) were synthesized according to published data [37] and were cloned into the pGPU6/GFP/Neo vector to express shRNA (short hairpin RNA). Following transfection with Lipofectamine 2000 (Invitrogen), we performed protein extraction, migration assay, or apoptosis assay. pGPU6/GFP/Neo-shNC (sense: 5′-CACCGTTCTCCGAACGTGTCACGTCAAGAGATTACGTGA-CACGTTCGGAGAATTTTTTG-3′ and antisense: 5′-GATCCAAAAAATTCTCCGAACGTGTCAC-GTAATCTCTTGACGT-GACACGTTCGGAGAAC) served as the negative control.

### 4.10. Migration Assay

To evaluate the migration of germ cells under the influence of sh*Prmt6*, we performed the following migration assay. First, 3–5 × 10^5^ cells were seeded in a 6-well plate and transfected with sh*Prmt6* or shNC using Lipofectamine 2000 once a monolayer was reached. With a 100-μL pipette tip, a scratch was made, and pure DMEM together with or without 10 nM testosterone was used to displace the old medium following a 6-h transfection. Cellular migration was tracked and recorded using a digital camera system at 0 h and 12 h after the wounds were created. The migration distance of sh*Prmt6* or shNC *versus* shNC’s were calculated and used as relative migration rates. Each well was observed at 4 points, and the experiments were repeated at least three times.

### 4.11. Flow Cytometry Analysis of Cell Apoptosis

For the apoptosis assay, germ cells were seeded in 6-well plates and transfected with sh*Prmt6* or shNC. Six hours after transfection, the medium was replaced, and the cells were treated with or without 10nM testosterone (T). After 48 h, the transfected cells were treated with Alexa Fluor^®^ 488 annexin V/Dead Cell Apoptosis Kit (Invitrogen). Adherent and floating cells were harvested, washed twice with cold PBS, and finally resuspended in 500 μL of 1× binding buffer. Subsequently, 5 μL of Annexin V-FITC and 3 μL of propidium iodide (PI) were added to the cell suspension; after a 15-min incubation at room temperature, the stained cells were analyzed within 1 h of staining by flow cytometry (Beckman Coulter, Miami, FL, USA) using an excitation of 488 nm according to the manufacturer’s instructions.

### 4.12. Statistical Analysis

All morphometric data were collected blindly. An independent-samples *t* test was used to compare the means between two groups using SPSS 17.0. Data are shown as the mean ± SD. Values of *p* < 0.05 were considered statistically significant.

## 5. Conclusions

In conclusion, we demonstrated that PRMT6 was regulated by the AR and that the expression of *Prmt6* mRNA and PRMT6 protein were up-regulated in ARKO mouse testes. Furthermore, *Prmt6* promoter-driven luciferase activity was significantly repressed by AR along with testosterone treatment, and PRMT6 could influence cell migration and apoptosis of germ cells. These data indicated that PRMT6 could play a potentially important role in spermatogenesis, even in male fertility. Further studies on the molecular mechanisms involved are needed to help us understand how PRMT6 is involved in spermatogenesis and how it is regulated by AR.
